# The Development of New Methodology for Determination of Vincristine (VCR) in Human Serum Using LC-MS/MS-Based Method for Medical Diagnostics

**DOI:** 10.3390/molecules27227945

**Published:** 2022-11-16

**Authors:** Kamil Jurowski, Łukasz Paprotny, Marcin Zakrzewski, Dorota Wianowska, Joanna Kasprzyk-Pochopień, Małgorzata Herman, Katarzyna Madej, Wojciech Piekoszewski, Tomasz Kubrak

**Affiliations:** 1 Laboratory of Innovative Toxicological Research and Analyses, Institute of Medical Studies, Medical College, Rzeszów University, Al. mjr. W. Kopisto 2a, 35-959 Rzeszow, Poland; 2 Department of Regulatory and Forensic Toxicology, Institute of Medical Expertises, Aleksandrowska 67/9, 91-205 Łódź, Poland; 3 Research and Development Centre, ALAB Laboratories, Ul. Ceramiczna 1, 20-150 Lublin, Poland; 4 Department of Chromatographic Methods, Faculty of Chemistry, Maria Curie-Skłodowska University, Pl. Maria Curie-Sklodowska 3, 20-031 Lublin, Poland; 5 Laboratory of High Resolution of Mass Spectrometry, Faculty of Chemistry, Jagiellonian University, R. Ingardena 3, 30-060 Krakow, Poland; 6 Department of Analytical Chemistry, Faculty of Chemistry, Jagiellonian University, R. Ingardena 3, 30-060 Krakow, Poland; 7 Department of Biochemistry and General Chemistry, Institute of Medical Studies, Medical College, Rzeszów University, Al. mjr. W. Kopisto 2a, 35-959 Rzeszow, Poland

**Keywords:** vincristine, oncovine, LC-MS/MS method, clinical practice

## Abstract

In this article, we have presented the development and validation of a rapid and sensitive reversed-phase liquid chromatography with tandem mass spectrometry (LC-MS/MS) method for the determination of vincristine (VCR) in patient serum samples. Chromatographic separation was achieved on a Kinetex^®^ (Singapore) column using a mobile phase consisting of 25 mM acetic acid and 0.3% formic acid (A) and methanol (B) in a gradient elution mode at a flow rate of 0.3 mL/min. The VCR and internal standard (vinblastine) were monitored using the multiple reaction monitoring mode under positive electrospray ionization. The lower limit of quantification (LLOQ) was 0.67 ng/mL, and the upper limit of quantification (ULOQ) was 250 ng/mL for VCR. The calculated values of LOD and LOQ for VCR were 0.075 and 0.228 ng/mL, respectively. The calibration curve was linear over the VCR concentration range of 1.0–250 ng/mL in serum. The intra- and inter-day precision and precision were within the generally accepted criteria for the bioanalytical method (<15%). The method was successfully applied to the analysis of serum samples in clinical practice.

## 1. Introduction

Vincristine (VCR) has been used in clinical practice since the early 1960s, and it has been approved by the Food and Drug Administration (FDA) as a drug against various types of cancer (e.g., Leukemia, Hodgkin’s lymphoma, lung cancer, breast cancer, Kaposi’s sarcoma, and testicular cancer) [1,2,3]. Today, about 60% of current anticancer medicinal products are derived from natural sources, including especially plants, marine organisms, and microorganisms. The molecules of the mentioned kind of pharmaceuticals can cause cell death through various mechanisms of actions, for example, DNA alkylation, inhibition of topoisomerase I or II, and additionally interference with neoplastic signal transduction or antiproliferative pathways [4,5]. The antimitotic effect is of great interest as it prevents abnormal growth of cancer cells by interfering with continued mitotic division. Reduces tumor growth by interfering with microtubules in the mitotic spindle and causes inhibition of normal hematopoiesis, followed by anemia, thrombocytopenia, and neutropenia [6,7]. The most common side effects monitored of VCR are neurotoxicity [8] and lipotoxicity as a reduction in the amount of adipose tissue resulting in peripheral and mainly symmetric sensorimotor neuropathy [9]. The studies conducted by Moore et al. (2011) on VCR pharmacodynamics and pharmacogenetics in children with cancer show the pharmacokinetic properties of this drug: volume of distribution (Vd, mean): 526 L/m^2^; clearance (Cl, median): 482 mL/min/m^2^, area under the curve (AUC, median): 49.7 g·h/L and biological half-life (t_1/2_, mean): 12.3 h [10]. It should be noted that the study described by Plasschaert et al. (2004) did not establish a significant relationship between pharmacokinetic parameters and the severity of neurological symptoms [11]. The study by Lavoie Smith et al. (2013) showed a significant correlation between higher severity of neurological symptoms and AUC for VCR [12]. Despite the extensive use of vincristine in the hematology and oncology of children and adults for more than three decades, it seems that so far, no reliable results have been presented regarding the correlation between pharmacokinetics and long-term anticancer effects of VCR.

One of the reasons may be the imperfection of the analytical methods used. It should be emphasized that since 2000, only a few papers reporting determination of VCR in human body fluids have been published. Liquid chromatography–mass spectroscopy was employed for the quantification of VCR in human plasma samples after solid phase extraction or liquid-liquid extraction [13,14,15]. Solvent bar micro-extraction combined with high-pressure liquid chromatography was used for pre-concentration and determination of trace amounts of VCR in body fluids such as plasma and urine [16]. The application of an electrochemical biosensor for the determination of VCR in human serum samples was also reported [17].

The aim of our work was to develop a fully validated LC-MS/MS method for rapid and selective determination of VCR in human serum samples for diagnostic purposes.

To the best of our knowledge, this paper is one of the very few studies on the use of a LC-MS/MS method for the monitoring of VCR in human serum.

## 2. Results and Discussion

### 2.1. LC–MS/MS Method Development

The LC-MS/MS method used for the analysis of vincristine has been checked and validated.

The development of the method began by checking two columns:

- Kinetex, grain 2.6 um, C18 100 A. 50 × 2.1 mm;
- Acquity BEH 1.7 μm, C18 50 × 2.1 mm

The Acquity column generated too much pressure; therefore, despite excellent chromatographic parameters, this election was rejected for further analysis and Kinetex 2.6 µm was used.

Then, experiments were conducted with different amounts of formic and acetic acids in the tested samples. Very good effects on the ionization and sensitivity of the tested samples were obtained for the addition of 25 mM acetic acid and 0.3% formic acid.

After many experiments in which the analytical method and sample preparation were optimized, the authors found that the method using the Kinetex column, grain 2.6 µm, C18 100 A. 50 × 2.1 mm, and the conditions described in the method will be validated and thoroughly checked for selectivity, precision, accuracy and other required parameters in accordance with the applicable standards.

The chromatographic analysis and ionization conditions for the experiments were performed using the Method Scout program by Shimadzu.

### 2.2. Assay Validation Results

The results obtained are shown in Supplementary Materials File S3. The maximum absolute value of the difference for IS retention times is 0.042 min and for VCR 0.051 min. Taking into account the accepted criterion indicated (0.1 min), this criterion was met. Furthermore, the acceptability criterion in this case was the relative signal difference, the difference in the ratio of ion signals determined for the test sample and calibration solutions against (S2/S1) Ref to 20% and the maximum was 4.5% (data not included).

According to the guidelines, the absence of interfering substances is determined by comparing the signals obtained during the analyte retention and the IS for the blank sample and the sample at the LLOQ level. The detector response during the analyte retention for the blank sample should constitute a maximum of 20% of the signal found in the LLOQ sample, and during IS retention, a maximum of 5% of the signal. In this case, 0.00% and 0.07%, respectively, were found. Thus, the acceptability criterion was met.

Examination of linearity of the chromatographic method for determination of VCR was carried out on a control serum enriched with the VCR in the required range of therapeutic serum concentration levels, i.e., 1.0–250 ng/mL. The minimal range should cover analyte concentrations from 50% (LLOQ, lower limit of quantitation) to 150% (ULOQ, upper limit of quantification). The linearity range of the developed method was 0.2–50 μg/mL and the determination coefficient (R) was >0.998 (R^2^ > 0.996). 

The calculated values of LOD and LOQ were 0.0752 and 0.2279 ng/mL of serum, respectively.

It was established on low (5.0 ng/mL), medium (75.0 ng/mL) and high concentration (250 μg/mL) levels of VCR in control serum samples. The accuracy was assessed on the basis of triplicate analysis of five control samples for each VCR concentration. The results obtained confirm the good precision of the VCR analysis at the three concentration levels mentioned in five different serum samples (−15.02 < Bias < 6.1). The acceptance criterion has been met [18].

In our studies, the repeatability and intermediate precision were determined. In general, repeatability means the precision under the prescribed operating conditions over a short period of time (usually during 1 day of analysis). To determine the repeatability of the VCR assay, five control serum samples, each enriched with analyte at three concentration levels (5.0, 75.0 and 250.0 ng/mL of serum), were subjected to triplicate chromatographic analysis. Intermediate precision is usually defined as the total precision under varied conditions in a laboratory (different days, different analysts, etc.). Intermediate precision was examined for three days. On each day five control serum samples, each enriched with analyte at three concentration levels (5.0, 10.0 and 15.0 ng/mL of serum), were subjected to triplicate chromatographic analysis. The analyzes were performed by different analyzes using different working standard solutions. The summary of intermediate precision of the VCR analysis in various fortified serum samples at three levels of analyte concentration is shown in Table 1.

The recovery of extraction was calculated as the percentage of the VCR response after sample preparation compared to the response of the analyte present in a standard solution at the known concentration. The extraction recoveries of VCR and IS from serum samples were determined at three concentration levels of the analyte, 5.0, 50.0 and 250 ng/mL of serum (concentration of IS was 25.0 ng/mL), in relation to appropriate concentrations of the analyte and IS in aqueous solutions.

Carryover usually refers to contamination of the sample being analyzed by the substance present in the autosampler loop after the previous analysis. Therefore, analyses were carried out using two alternately dosed solutions, the first of which was the calibration solution with the highest concentration of the VCR and the other a blank sample containing neither the analyte nor the internal standard. The lack of carryover was evidenced by the absence of peaks in the chromatogram corresponding to a blank sample solution, which was dosed directly after dosing a calibration solution with the highest concentration of the VCR.

In general, ruggedness should be understood as a measure for the susceptibility of a method to small changes (e.g., pH values, temperature, mobile phase composition, etc.) that might occur during routine analysis. In our study, changes in mobile phase composition (change in phase B participation: ±5%), column temperature (±15%) and flow rate (±10%) were examined. For this task, a blank serum sample enriched with 5.0 μg/mL of VCR and 20 μg/mL of IS was subjected to analyzes under the conditions assumed above. Under the above-mentioned changes to the experimental conditions, the acceptance criterion was met and the VCR concentration ranged between 97 and 103% in relation to the concentration read under the initial conditions.

Stability usually means the chemical stability of an analyte in a given matrix under specific conditions for given time intervals. In our study, the stability of VCR in a solution was estimated as the recovery determined for test solutions in relation to the freshly prepared solution within ±2% of the initial value. The stability of a serum sample spiked with VCR (5.0 ng/mL) and IS (20 ng/mL) was tested under three conditions:

(I) Stored for 120 h in the autosampler;
(II) Stored for a period of 120 h exposed to light at room temperature in the laboratory;
(III) Subjected to freezing and thawing several times (five cycles).

A serum sample spiked with VCR and IS was stable under the tested conditions (0.07 < CV < 0.05), so the criterion was met.

### 2.3. Comparison of Our Method with the Methods of Other Authors

Until now, there have only been a few chromatographic methods based on mass spectrometric detection to analyze VCR in serum [14,15] which can be useful in modern clinical practice. Guo et al. [13] determined VCR in mouse serum, the advantage of the method allowed the quantification of VCR in multiple serum samples obtained from a single mouse, which allowed an accurate estimation of its pharmacokinetic properties. However, recovery was very low (57%), and the method was validated at serum VCR concentrations of 0.01 to 2.0 µg/mL. On the other hand, an analytical methodology based on electrospray ionization and high-performance liquid chromatography/tandem mass spectrometry (LC/ESI-MS/MS) was developed by Dennsion et al. [15] to quantify VCR and M1 (the CYP3A-mediated metabolite of VCR) in human serum. The concentration limits of quantification and detection were 12 pg/mL and 6 pg/mL, respectively. Furthermore, Corona et al. [14] described a simple method for the determination of VCR in human serum by LC/MS/MS with atmospheric pressure chemical ionization using online solid phase extraction. The recovery for VCR was within 90%, the intra-day and inter-day assay precision ranged from 1.2% to 6.8% RSD, while the mean percentage deviation from the nominal value ranged from 0.01% to 6.1%. The proposed assay was found to be suitable for pharmacokinetic investigations and clinical monitoring of therapeutic drugs, especially in pediatric cancer patients. However, the limited serum sample required (100 µL) can be considered as a disadvantage in modern clinical practice, especially in pediatric patients’ studies. Therefore, based on the results of the mentioned articles, we developed a highly sensitive, selective, rapid, and robust method using LC–MS/MS instrumentation for VCR determination in human serum.

### 2.4. Application of the Validated Method to Analysis of Clinical Samples

The validated method was applied to quantify vincristine in plasma taken from twenty patients (*n* = 20 samples), receiving a mean dose of (1.2 ± 0.4) mg/m^2^ of vincristine by 5 min intravenous injection (based on [19]). The results of the analysis of plasma samples for the determination of VRC are presented in Table 2. The observed variation is about 2.3%. This is a normal observation because all patients received the same amount of VCR according to clinical data in the literature [19].

## 3. Materials and Methods

### 3.1. Chemicals

Standards substances of vincristine sulfate and vinblastine sulphate used as an internal standard (IS) were obtained from Sigma Aldrich Corp. (St. Louis, MO, USA) and ChromaDex, Inc. (Irvine, CA, USA). The methanolic stock solutions were stored at 18 °C and brought to room temperature prior to use. LC-MS ultra-grade methanol was purchased from Honeywell LabReady™. Formic acid (98%, *w*/*w*) was purchased from VWR Inter-national LLC. Zinc sulfate heptahydrate (HPLC purity) from Sigma-Aldrich was applied. The water used in the entire analysis was prepared using a MilliQ water purification system from Millipore.

### 3.2. Samples

For the validation of the method, control human serum (conserved with heparin) was applied. The expired serum was purchased from a local blood bank in Lublin (Poland). To verify the developed method for therapeutic drug monitoring (TDM), plasma samples were collected from VCR-treated patients.

Calibration and quality control (QC) serum samples were prepared by spiking appropriate working solutions with drug-free human serum. 

Control samples (QS) were prepared by fortification of drug-free serum with the analyte at low (5 ng/mL), medium (50 ng/mL) and high (250 ng/mL) concentration levels with the addition of the IS at the concentration 25 ng/mL). The volumetric ratios applied for the preparation of calibration solutions and QS are presented in Supplementary Materials File S2.

### 3.3. LC-MS/MS Conditions

A Shimadzu Nexera X2 UHPLC system equipped with a Shimadzu LCMS-8050 triple quadrupole mass spectrometer (Q-Q-Q-MS) detector, autosampler SIL-30ACXR; two LC-30AD pumps, DGU-20A5R degasser; CTO-20AC furnace and LCMS-8050 detector was applied.

Chromatographic analysis was performed on the Kinetex^®^ column (50 × 2,1 mm, 2.6 µm) using gradient elution with a mobile phase consisting of 25 mM acetic acid and 0.3% formic acid in water (A) and methanol (B), with a flow rate of 0.3 mL/min. The column temperature was maintained at 45 °C. An electrospray ionization (ESI) source operating in the positive ionization mode was used for multiple reaction monitoring (MRM). The optimized MS/MS conditions for quantification of VCR and vinblastine are shown in the Supplementary Materials File S1.

### 3.4. Calibration Strategy, Quality Control, and Statistical Analysis

A series of standard VCR working solutions ranging from 50 to 5000 ng/mL and a standard IS solution (2.5 µg/mL) were obtained by a suitable dilution of the appropriate stock solutions with methanol and methanol/water (50/50, *v*/*v*), respectively.

The selective/specific analytical method should allow the resolution and detection of the drug of interest and IS in the presence of possible co-administered pharmaceuticals and co-eluting endogenous compounds in the matrix [18].

The confirmation of the specificity of the assay was based on the analysis of:

(1) The constancy of the retention times of vinblastine and the IS in test samples against calibration solutions;
(2) The constancy of the ratio of fragmentation ion signals of both compounds in the tested samples in relation to the calibration solutions;
(3) The ratio of the signals obtained during the retention of the analyte and the internal standard for blank samples (*n* = 5) versus the sample at the LLOQ level.

In general, linearity of an analytical method means a direct proportional response of the results obtained to the analyte concentrations in an appropriate calibration set. This parameter was estimated for a blank serum sample and six concentration levels of spiked control serum samples with VCR. A measurement was made for each concentration level in triplicate.

The limits of detection (LOD) and quantification (LOQ) were determined from Equations (1) and (2), respectively:

LOD = (3.3 × SD)/a(1)

LOQ = (10 × SD)/a(2)

where:

SD—standard deviation of the regression line;

a—the directional coefficient of the calibration curve.

In general, the accuracy of a method is affected by systematic and random error components. Trueness is related to the closeness of agreement between the conventionally accepted value in comparison to the mean experimental value. Usually, this parameter is estimated as a percentage deviation from the accepted reference value (Bias) [18].

Precision can be defined as a degree of scatter between a series of measurements from multiple sampling of the same homogeneous sample under the same experimental conditions. In most cases, this parameter is expressed as imprecision, and given as relative standard deviation (RSD, CV). It should be underlined that precision may be considered as repeatability (also known as intra-assay, within-run precision or within-day precision) or intermediate precision (inter-assay, between-run or between-day precision).

The raw results obtained were analyzed applying statistical software: Excel 2010 (Microsoft Office), LabSolutions-LC solutions (Shimadzu, Kyoto, Japan), and Origin 2021 Pro the Ultimate Software for Graphing and Analysis (OriginLab Corporation, One Roundhouse Plaza, Suite 303, Northampton, MA, USA) licensed by the Jagiellonian University in Krakow. Data processing, all preliminary calculations and storage of obtained data at laboratory stage were performed using data acquisition software LabSolutions-LC solutions (Shimadzu). The results of five independent replicates were expressed as the mean ± standard deviation. All graphs were plotted using Origin 2021 Pro.

### 3.5. Sample Preparation

Until analysis, human serum/plasma samples were centrifuged for 10 min at 12,500× *g*. In the first step, a 200 μL aliquot of centrifuged sample was placed in an Eppendorf tube, then 20 μL of the internal standard solution (2.5 μg/L in methanol/water; 50/50; *v*/*v*) and 80 μL of zinc sulphate solution (in methanol, 0.2 M) were added. In the mixture next step, the obtained in the tube was vortexed for 60 s and centrifuged for 10 min at 12,500× *g*. Finally, 200 μL of a supernatant was taken to an autosampler vial and injected into the chromatographic column.

## 4. Conclusions

The idea of the research was the development and validation of a rapid and sensitive LC-MS/MS-based methodology for the determination of VCR in patient serum samples. The lower limit of quantification (LLOQ) was 0.670 ng/mL, and the upper limit of quantification (ULOQ) was 250 ng/mL of VCR in serum. The calculated values of LOD and LOQ were 0.0752 and 0.2279 ng/mL of serum, respectively. The calibration curve was linear over the VCR concentration range of 1.0–250 ng/mL in serum (pharmacokinetics profile of VCR). Intra-day and inter-day precision were within the generally accepted criteria for the bioanalytical method (<15%). 

Its usefulness for clinical practice was checked by applying it to the analysis of human plasma samples (*n* = 20 patients) treated with VCR (see Table 2). The observed variation is about 2.3%, but this is normal based on clinical data [19]. It can be concluded that the proposed methodology is appropriate for the determination of VCR in serum for drug monitoring purposes, as well as for pharmacokinetic studies.

It should be underlined that our methodology was successfully applied to clinical practice for important clinical problems.

## Figures and Tables

**Table 1:** Intermediate precision of vincristine analysis in various fortified serum samples at three levels of analyte concentration.

Series	Level of DSP	C_exp_ [ng/mL]	SD [ng/mL]	CV
	[ng/mL]	*n* = 5	*n* = 5	[%]
	3.3	3.067	0.081	2.65
I	50.17	44.301	1.208	2.74
	167.23	173.805	5.438	3.14
	3.3	3.136	0.124	4.0
II	50.17	44.72	0.49	1.10
	167.23	172.559	5.937	3.41

**Table 2:** Results for VRC in patients’ plasma by the developed method.

Patient Number	Concentration Level of VRC, ng/mL
1	70.02
2	75.56
3	74.05
4	73.21
5	73.17
6	52.39
7	51.75
8	49.15
9	49.63
10	52.18
11	51.40
12	56.70
13	103.05
14	88.00
15	83.45
16	85.68
17	79.63
18	58.34
19	54.49
20	61.55

## Data Availability

Not applicable.

## Supplementary Materials

The following supporting information can be downloaded at: https://www.mdpi.com/article/10.3390/molecules27227945/s1, File S1: The optimized MS/MS conditions for quantification of VCR and Vinblastine; File S2: Volumetric ratios used to prepare working calibration solutions. Volumetric ratios used for the preparation of control samples (QS); File S3: Retention times of compounds in calibration solutions. The differences in retention times of compounds in the test sample and calibration solutions.

## Sample Availability

Not available.
