# Supplementary material for: The Development of New Methodology for Determination of Vincristine (VCR) in Human Serum Using LC-MS/MS-Based Method for Medical Diagnostics
Source: Molecules. 2022 Nov 16;27(22):7945. doi: 10.3390/molecules27227945 (PMC9694046; doi:10.3390/molecules27227945)
Supplement: Supplementary file 1 [file molecules-27-07945-s001.zip › Supplementary Materials S1.pdf]

Supplementary Materials S1 (SMS1)

The optimized MS/MS conditions for quantification of VCR and Vinblastine:

Vinblastine:

811,40 -> 751,4 m/z: CE -44 (quantitative analysis)

811,70 -> 224,20 m/z; CE -46 (qualitative analysis)

Vincristine:

825,70 -> 807,60 m/z; CE: -40
