# Supplementary material for: The Development of New Methodology for Determination of Vincristine (VCR) in Human Serum Using LC-MS/MS-Based Method for Medical Diagnostics
Source: Molecules. 2022 Nov 16;27(22):7945. doi: 10.3390/molecules27227945 (PMC9694046; doi:10.3390/molecules27227945)
Supplement: Supplementary file 1 [file molecules-27-07945-s001.zip › Supplementary Materials S2.pdf]

Supplementary Materials S2 (SMS2)

Volumetric ratios used to prepare working calibration solutions

Primary stock solution	Volume of Vinblastine, ng/mL	Volume of plasma, µL	Volume of Vinblastine solution, µL	Volume of Vinblastine stock solution, µL	Volume of $ZnSO_4$ solution in methanol, µL	Sum of sample volume, µL	Concentration of Vinblastine in plasma, ng/mL	Concentration of IS in plasma, ng/mL	Concentration of Vinblastine in solution, ng/mL	Concentration of IS in solution, ng/mL
0	-	200	0	25	80	300	0	0	0	16.73
1	50	200	5	25	75	300	1.0	25	0.67	16.73
2	50	200	25	25	55	300	5.0	25	3.33	16.73
3	500	200	10	25	70	300	25.1	25	16.72	16.73
4	500	200	20	25	60	300	50.2	25	33.45	16.73
5	5000	200	3	25	77	300	75.3	25	50.17	16.73
6	5000	200	5	25	75	300	125.4	25	83.62	16.73
7	5000	200	10	25	70	300	250.9	25	167.23	16.73

Volumetric ratios used for the preparation of control samples (QS)

Solution	VCR solution, ng/mL	Calibration solution	Volume of plasma, µL	Volume of VCR stock solution, µL	Volume of IS stock solution, µL	Volume of $ZnSO_4$ solution in methanol, µL	Sum of sample volume, µL	Concentration of VCR in plasma, ng/mL	Concentration of IS in plasma, ng/mL	Concentration of VCR in solution, ng/mL	Concentration of IS in solution, ng/mL
1	50	1	200	5	25	75	300	5	25	0.67	16.73
2	500	2	200	20	25	46	300	50	25	33.45	16.73
3	5000	3	200	10	25	38	300	250	25	167.23	16.73
