# Supplementary material for: The Development of New Methodology for Determination of Vincristine (VCR) in Human Serum Using LC-MS/MS-Based Method for Medical Diagnostics
Source: Molecules. 2022 Nov 16;27(22):7945. doi: 10.3390/molecules27227945 (PMC9694046; doi:10.3390/molecules27227945)
Supplement: Supplementary file 1 [file molecules-27-07945-s001.zip › Supplementary Materials S3.pdf]

Supplementary Materials S3 (SMS3).

Retention times of compounds in calibration solutions

Calibration solution	VCR<br>$t_{R\ cal}$, min	IS<br>$t_{R\ cal}$, min
1	4.210	4.010
2	4.209	4.026
3	4.209	4.020
4	4.209	4.025
5	4.209	4.015
6	4.210	4.013
average t_R:	4.209	4.018

The differences in retention times of compounds in the test sample and calibration solutions

n	VCR		IS	
	t_R	t_R - t_{Rcal}	t_R	t_R - t_{Rcal}
P_1	4.158	-0.051	4.050	0.032
P_2	4.189	-0.020	4.060	0.042
P_3	4.178	-0.031	4.060	0.042
P_4	4.189	-0.020	4.060	0.042
P_5	4.186	-0.023	4.050	0.032
P_6	4.189	-0.020	4.050	0.032
